# Pyrethroid insecticides maintain repellent effect on knock-down resistant populations of *Aedes aegypti* mosquitoes

**DOI:** 10.1371/journal.pone.0196410

**Published:** 2018-05-15

**Authors:** Natalie M. Bowman, Kristin Akialis, Grayson Cave, Roberto Barrera, Charles S. Apperson, Steven R. Meshnick

**Affiliations:** 1 Department of Medicine, Division of Infectious Diseases, University of North Carolina, Chapel Hill, North Carolina, United States of America; 2 Department of Epidemiology, University of North Carolina Gillings School of Global Public Health, Chapel Hill, North Carolina, United States of America; 3 Department of Entomology and Plant Pathology, North Carolina State University, Raleigh, North Carolina, United States of America; 4 Centers for Disease Control and Prevention, San Juan, Puerto Rico, United States of America; University of Crete, GREECE

## Abstract

Pyrethroid-treated clothing is commonly worn for protection against mosquitoes; pyrethroids are both insecticides and repellents. Pyrethroid resistance has become increasingly common in *Aedes aegypti*, the vector of dengue, Zika, and other arboviruses, but it is not clear whether resistance is associated with reductions in repellency. In order to determine whether long-lasting permethrin impregnated (LLPI) clothing is protective, we used *Aedes aegypti* from New Orleans, LA (pyrethroid-sensitive) and San Juan, PR (resistant) to measure both lethality and repellency. PCR and Sanger sequencing were used to confirm resistance status by detecting mutations in the *kdr* gene at positions 1016 and 1534. Arm-in-cage trials of 100 *Aedes aegypti* females from both populations were performed for 10 minutes to bare arm or an arm clothed in untreated military camouflage or military camouflage impregnated with deltamethrin, permethrin, or etofenprox. Trials were repeated 4–5 times on different days. Number of landings, number of blood meals, and immediate and 24-hour mortality were recorded. Mortality was extremely low in all trials. Compared to untreated cloth, mosquitoes demonstrated a trend towards a 2%-63% reduction in landings and a statistically significant 78–100% reduction in blood feeding on pyrethroid-treated cloth for most insecticides. Effects were observed in both pyrethroid-sensitive and pyrethroid-resistant mosquito populations. Our data show that *kdr* mutations are associated with pyrethroid resistance but are likely not the only contributors. Pyrethroids appear to maintain repellent effect against resistant mosquitoes. This finding suggests that even in places where pyrethroid resistance is widespread, permethrin still has a role for use as a repellent on clothing to protect against mosquito bites.

## Introduction

*Aedes aegypti* mosquitoes are the vectors of several important human infections including dengue, Zika, chikungunya, and yellow fever, which affect hundreds of millions of people each year globally in tropical and subtropical regions, causing a burden of disease equal to more than four million disability-adjusted life years in 2013.[1,2] Vector control remains a key component in preventing these illnesses, and pyrethroid insecticides have been among the most widely used vector control tools because of their efficacy and favorable mammalian toxicity profile. Pyrethroid insecticides are used for indoor and outdoor spraying and currently are the only class used for treating bed nets;[3] additionally, they are gaining popularity for use in clothing.[4–8] The widespread use of pyrethroids, however, has led to increasing resistance to some or all pyrethroids in many *Aedes* populations.

Pyrethroids exert their insecticidal effect on the voltage-gated sodium channel (VGSC) located on the membrane of neurons. When pyrethroids bind an open channel, they prevent its closure, thus prolonging the action potential and resulting in the insect’s rapid paralysis, known as “knockdown” or kdr, and death. Several point mutations in the *Aedes aegypi* VGSC have been identified, though only a few have been definitively linked to a resistant phenotype.[9–14] Two SNPs in domain II and domain III, at positions 1016 and 1534, respectively, have been well studied. In Latin American *Ae*. *aegypti* populations, isoleucine replaces valine at position 1016 and cysteine replaces phenylalanine at position 1534. Both mutations have been associated with high-level kdr resistance, though the evolution of and interaction between these two mutations are not well-understood.[15] While there is substantial geographic variability in the distribution of particular *kdr* mutations, this mechanism of pyrethroid resistance has become widespread,[16] compromising the utility of these insecticides for vector control.

In addition to their insecticidal effect, pyrethroids have both spatial repellent and contact irritant effects,[17–19] though excitorepellency is probably the most important effect when pyrethroids are used on clothing. Limited evidence suggests that pyrethroid-resistant populations may actually be more strongly repelled by pyrethroids and other repellents than fully susceptible mosquitoes.[20–22] If pyrethroids have potent repellent effect against both susceptible and resistant *Ae*. *aegypti*, then they may remain useful tools for personal protection against mosquitoes despite the increasing prevalence of pyrethroid resistance worldwide.

In this study, we tested repellent activity of military-grade cloth factory-impregnated with three different pyrethroid insecticides (permethrin, deltamethrin, and etofenprox) against susceptible and resistant strains of *Ae*. *aegypti*. Genetic markers of resistance, specifically *kdr* mutations, were documented in these same populations. Our results demonstrate that pyrethroid-impregnated cloth can exhibit repellent activity even against pyrethroid-resistant mosquito populations.

## Materials and methods

### Mosquito colony origin and maintenance

Susceptible *Aedes aegypti* eggs were collected in New Orleans, LA in 2008,[23,24] and generation F12-13 mosquitoes (NO mosquitoes) were used in tests. Resistant *Ae*. *aegypti* eggs were collected from San Juan, Puerto Rico in 2016,[25] and generation F1-2 adult females (PR mosquitoes) were used in bioassays. Although mosquito colonies were derived from eggs collected in different years, all experiments were performed over a brief period in the fall and winter of 2016–2017 using adult mosquitoes descended from the original collected mosquito eggs. Mosquito colonies were maintained as described previously.[23,26] Colonies were maintained in separate containers at approximately 26°C and at a relative humidity of ≈75% under a photo regime of 14 light:10 dark hours. The light phase included two 30-min crepuscular periods (40-watt incandescent bulb) daily. Larvae were fed a 2:1 mixture of liver powder:baker’s yeast on a standardized schedule. Adults were housed in 30 × 30 × 30-cm Plexiglas cages fitted with cotton surgical stocking tops and fed 10% sucrose solution *ad libitum*.[26] Adult females were not permitted to blood-feed prior to experiments.

### Mosquito DNA extraction

*Ae*. *aegypti* mosquitoes used for *kdr* genotyping were field collected from Puerto Rico (resistant) and New Orleans (susceptible). Mosquito DNA was extracted using published protocols.[27,28] Briefly, individual mosquitoes were manually homogenized in 100 μl PBS and 10 μl 10% saponin solution. The mosquito homogenate was incubated for 20 minutes at room temperature then centrifuged at 20,000 x g for 2 minutes. The pellet was resuspended in 200 μl PBS and centrifuged again; the supernatant was removed and the pellet was resuspended in 75 μl deionized water and 25 μl Chelex then gently vortexed and incubated in a 95–99°C water bath for 13 minutes. Finally, the solution was centrifuged for 2 minutes at 20,000 x g and the supernatant containing gDNA was transferred to a clean tube and stored at -20°C.

### Gene amplification and sequencing

The voltage-gated sodium channel (*kdr*) gene from *Ae*. *aegypti* was amplified using a protocol adapted from Sayano, *et al*.[29] The IIS6 and IIIS6 regions of the *kdr* gene were amplified using the following primers: KasaikdraegSCF20 5’-GACAATGTGGATCGCTTCCC-3’ and KasaiddraegSCR21 5’-GCAATCTGGCTTGTTAACTTG-3’ (domain II) and KasaikdraegSCF7 5’-GAGAACTCGCCGATGAACTT-3’ and KasaikdraegSCR7 5’GACGACGAAATCGAACAGGT-3’ (domain III) and FastStart High Fidelity PCR system (Roche, Mannheim, Germany). PCR reactions contained 5 μl template DNA in a mixture of 12.75 μl water, 2.5 μl 10X FastStart High Fidelity Reaction Buffer, 0.25 μl FastStart HighFidelity Enzyme Blend, 0.5 μl 10mM dNTPs, 2 μl forward primer, and 2 μl reverse primer. Amplification was performed under the following conditions: 95°C for 2 minutes; 35 cycles of 95°C for 30 seconds, 58°C for 30 seconds, and 72°C for 30 seconds; 72°C for 5 minutes and a 4°C hold.

Sanger sequencing was then performed on PCR products using the primers aegSCF3 5’-GTGGAACTTCACCGACTTCA-3’ for domain II and aegSCR8 5’-TAGCTTTCAGCGGCTTCTTC-3’ for domain III (Eton). Sequences were aligned with published sequences (Genbank IDs KJ957878-KJ957893 and KF537414-15, JF4796611-12, and JX275501) using Mega software (www.megasoftware.net).[30]

### Arm-in-cage testing

Mosquito knock-down testing was performed using United States military cloth (6.8 oz/yd^2^ 50% nylon/50% cotton rip stop printed with current ACU camouflage pattern) either untreated or chemically impregnated with 0.52% insecticide (deltamethrin, permethrin, or etofenprox) by weight as an even application, or by bare arm. The arm was cleaned with 70% ethanol and allowed to dry between trials. The forearm of a volunteer was covered with a piece of the candidate cloth. A cover constructed from white EPDM (60 mil) rubber roofing membrane was attached over the cloth with Velcro straps. The cover had an opening (33 x 150 mm) that exposed the candidate cloth. The volunteer’s hand and wrist were covered with a nitrile glove. At the start of each test, the volunteer inserted his forearm into a Plexiglas cage (30 cm on each side) through a cloth sleeve. The cage contained exactly one hundred 5–7 day old female *Aedes aegypti* mosquitoes (either NO or PR strain). In a few cases, 1 or 2 mosquitoes died before exposure to the clothing; in these cases, analyses were adjusted for the slight decrease in number of exposed mosquitoes by dividing by counts by the proportion alive at the beginning of each trial. Each bioassay was conducted for 10 minutes during which the number of mosquitoes landing on the cloth was counted. A mosquito was counted as landing if it touched the cloth for at least 2 seconds. The number of mosquitoes that probed the cloth and obtained a blood meal was also counted. Mosquito mortality was determined 24 hours after each bioassay was terminated. Tests were repeated for each fabric on four different days for New Orleans mosquitoes and on five different days for Puerto Rico mosquitoes, with one replicate per day. Untreated cloth was used as the control each day. Bare arm control was used in two trials to assess any repellent effect of the untreated cloth by itself. All experiments were performed between October 2016 and February 2017. Temperature for the trials ranged from 25.7 to 28.1°C and relative humidity ranged from 67.4 to 88.0%. Two male human subjects were used for the test but were not systematically rotated. Subjects provided both written and verbal informed consent (North Carolina State University IRB protocol 2925, annual renewal approved 6/21/2017).

### Statistical analyses

Associations between mosquito strain (NO or PR) and the presence of *kdr* mutations were assessed using Chi-squared tests. Insecticidal effect was defined as the increase in 24-hour mortality between control (untreated) and treated cloth. Repellency was measured two ways: 1) reduction in number of landings and 2) reduction in number of blood meals between control and treatment tests performed on the same day. These numbers were standardized to the number of live mosquitoes present at the beginning of a given test. Results were compared using Student’s *t*-test, Kruskal-Wallis equality-of-populations rank test, Wilcoxon rank sum test (Mann Whitney two sample statistic), and Wilcoxon matched pairs signed rank test with untreated cloth as the referent. Repellency was expressed as reduction in landing or blood feeding behavior calculated by the following equation:

%repellency=100×(controlcount−treatmentcount)/controlcount (1)

for landings or blood meals. The control count used for each calculation was the one performed on the same day as the treatment count.

### Ethics

Protocol 2925 was approved by the North Carolina State University Institutional Review Board. The approved procedure involved arm-in-cage studies of pyrethroid insecticides against *Aedes aegypti* mosquitoes. This procedure has been reviewed and approved by the North Carolina State University Institutional Review Board (IRB Protocol 2925, annual renewal approved 6/21/2017). Both verbal and written informed consent were obtained from study participants.

## Results

The gene for the *Ae*. *aegypti* voltage-gated sodium channel (*kdr*) was amplified from 19 mosquitoes from the pyrethroid-susceptible New Orleans population (NO) and 22 mosquitoes from the pyrethroid-resistance Puerto Rico population (PR). The *kdr* gene was successfully sequenced at domain II (1016 location) from 19 NO mosquitoes and 18 PR mosquitoes and at domain III (1534 location) from 19 NO and 15 PR mosquitoes. Results are summarized in Table 1. Heterozygosity was common in both populations at both SNPs, though both *kdr* mutations were more common in the PR mosquitoes, as expected. While PR mosquitoes were more likely to have either *kdr* mutation, this relationship was only statistically significant for the presence of at least one 1016 mutation (OR 13.71, 95% CI 2.04, 145.62). No mosquitoes were homozygous for *kdr* mutations at both SNPs. All PR mosquitoes had at least one *kdr* mutation, while only 11/19 (58%) of NO mosquitoes had at least one *kdr* mutation (p = 0.01). PR mosquitoes were more likely to have both mutations, though this was not statistically significant (p = 0.1). Thus, kdr alleles were present in both populations but were found in all members of the PR population.

**Table 1:** *kdr* (voltage gated sodium channel) gene sequencing from susceptible and resistant mosquito populations.

	Wild type	Heterozygous	kdr	No sequence	No kdr	1 kdr	Both kdr
**Susceptible (NO)**					8	6	5
1016	12	6	1	0			
1534	10	9	0	0			
**Resistant (PR)**					0	5	6
1016	2	14	2	4			
1534	3	9	3	7			

Arm-in-cage tests were performed on nine different days. There was wide variability in biting behavior between days despite the temperature- and humidity-controlled environment. In a multivariate linear regression model, neither temperature nor relative humidity was associated with landing and feeding behavior, adjusted for mosquito strain and clothing treatment (untreated vs. treated).

**Fig 1:**
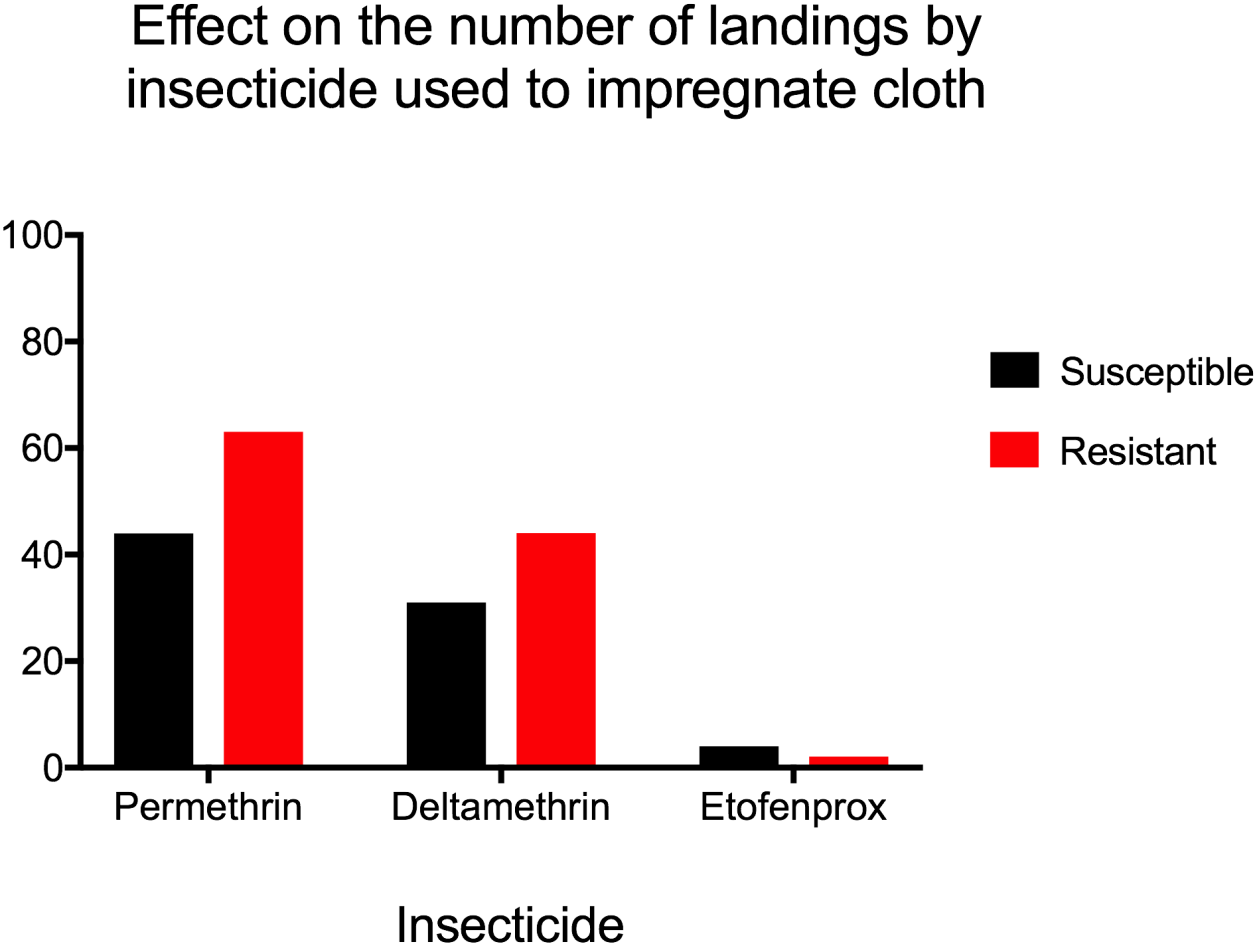
Effect on the number of landings by insecticide used to impregnate cloth. Referent group is untreated cloth. All three insecticides reduced number of landings.

**Fig 2:**
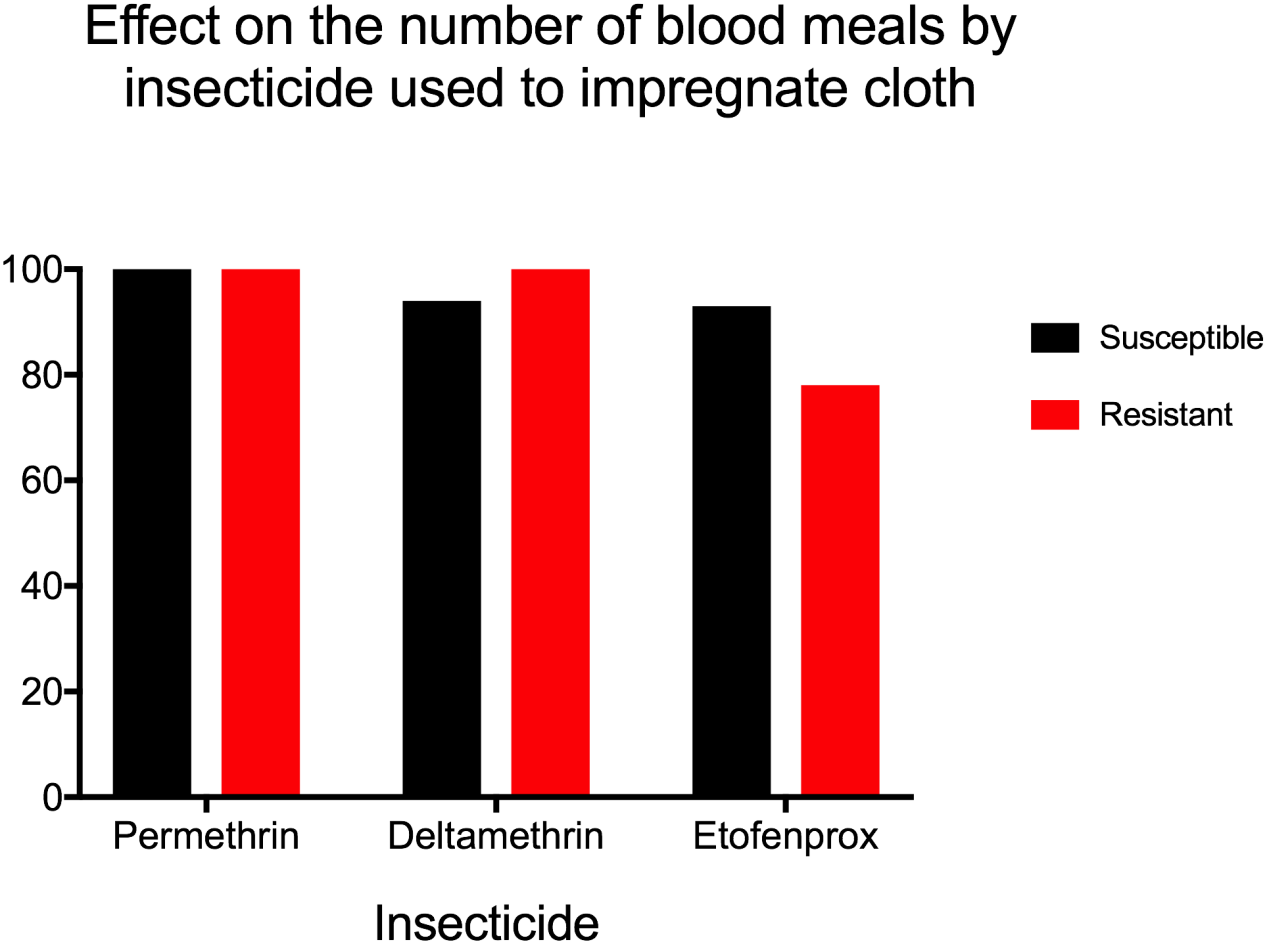
Effect on the number of blood meals by insecticide used to impregnate cloth. Referent group is untreated cloth. All three insecticides reduced the number of mosquitoes taking a blood meal by 78–100%, with similar results for sensitive (NO) and resistant (PR) mosquito populations.

Compared to bare arm, untreated cloth protected against landing and blood feeding (p = 0.03 for both by Wilcoxon rank-sum test). Untreated cloth was used as a control for the evaluation of pyrethroid-treated fabric in arm-in-cage tests, shown in Table 2. By Kruskal-Wallis test, there was no statistically significant difference in reduction in landings between clothing treatments for the NO mosquito strain (p = 0.2), but insecticides did have a statistically significant effect on landing for the resistant PR strain (p = 0.02). The reduction in number of blood meals taken was significantly different for PR mosquitoes (p = 0.02), with a trend towards significance in the NO group (p = 0.06). When fabrics impregnated with different insecticides were compared to untreated cloth using pair-wise Wilcoxon signed rank test, we observed a protective effect of pyrethroid-treated clothing against landing and blood-feeding, but the difference did not reach statistical significance in most cases (Table 2). While all treated clothing reduced the number of landings compared to untreated clothing (Figs 1 and 2), this effect was only significant for the reduction in landings by permethrin. However, the reduction in blood meals compared to untreated control was statistically significant in most trials, with the exception of deltamethrin in the NO group (Table 2). Mosquito mortality at 24 hours was surprisingly low in all trials (0–11%), with no significant differences between resistant and susceptible populations or between untreated and insecticide-impregnated cloth.

**Table 2:** Results of arm-in-cage testing for pyrethroid-susceptible (NO) and pyrethroid-resistant (PR) mosquitoes. Results are expressed as the median (range) of the trials. Four replications were performed for trials of NO mosquitoes. Fiver replications were performed for PR mosquitoes, with the exception of 4 trials for deltamethrin. All % reduction measurements are made compared to same-day untreated cloth controls.

	Landings	% reduction in landings	Blood meals	% reduction in blood meals	% landings that blood fed	% reduction in blood meals/landing	24-hour mortality (%)
**Susceptible (NO)**							
Untreated	273 (48–442)	.	5.5 (1–14)	.	2.1 (1.0–4.0)	.	0.5 (0–2)
Permethrin	129 (19–227)^#^	44 (34–60)^†^	0 (0–1)^#^	100 (89–100)*	0 (0–0.4)^#^	100 (78–100)*	0 (0–3)
Deltamethrin	135 (54–225)	31 (-159-88)	0.5 (0–1)	94 (-1-100)	0.4 (0–1.9)^#^	80 (9–100)*	1.5 (0–4)
Etofenprox	256 (44–368)	4 (-88-63)	1 (0–3)^#^	93 (67–100)*	0.3 (0–1.9)^#^	93 (9–100)*	0 (0–4)
**Resistant (PR)**							
Untreated	200 (127–357)	.	4 (0–15)	.	1.7 (0–7.9)	.	0 (0–1)
Permethrin	78 (38–125)^†^	63 (46–78)^#^	0 (0–0)^#^	100 (100–100)^†^	0 (0–0)^#^	100 (100–100)^†^	0 (0–7)
Deltamethrin	107 (62–213)^#^	44 (32–71)^#^	0 (0–1)^#^	100 (66–100)*	0 (0–1.0)	100 (-17, 100)	0 (0–3.1)
Etofenprox	182 (22–308)	2 (-4-83)	1 (0–3)^#^	78 (0–100)*	0.3 (0–2.7)	78 (-221-100)	0 (0–11)

^†^p≤0.01

*p≤0.05

^#^p≤0.10

There was not a significant difference in reduction of mosquito landings or blood meals by deltamethrin or etofenprox between strains; however; permethrin caused a significantly stronger reduction in landing for the PR mosquitoes (p = 0.05). Additionally, the effect of pyrethroid-treated cloth was even stronger in reducing blood feeding than in preventing landings, almost completely abolishing blood feeding activity in the case of permethrin and deltamethrin (Table 2, Figs 1 and 2).

## Discussion

Here we demonstrate that even in the absence of significant insecticidal activity, clothing impregnated with the pyrethroids permethrin, deltamethrin, and etofenprox exhibited the ability to repel *Aedes aegypti* mosquitoes. This effect was observed for both pyrethroid-sensitive and pyrethroid-resistant strains of mosquito.

Mosquito resistance to pyrethroids has become a global problem. Of the major mosquito-borne diseases, only yellow fever has a highly effective vaccine available. Thus, vector control remains the cornerstone of interventions to prevent arboviruses and malaria. As illustrated by the incomplete protection afforded by currently available vaccines for dengue and malaria [31–37] and by the vaccine shortages complicating control of the recent yellow fever outbreaks in Brazil, Angola, and the Democratic Republic of Congo,[38–43] vector control remains a crucial public health intervention for prevention and control of mosquito-borne infections. Because of pyrethroids’ safety and potency, they are widely used as adulticides, leading over time to the development of high levels of pyrethroid resistance in many areas most affected by arboviruses and malaria.

Pyrethroids are also widely used for community and household protection via indoor and outdoor area spraying and for personal protection through the use of insecticide impregnated for bed nets and clothing. While area spraying’s effect is mainly realized by reduction in the population of mosquitoes in the area, pyrethroid-treated bed nets and clothing can provide complete personal protection from vector-borne disease simply by preventing bites. Even if mosquitoes are pyrethroid-resistant, bed nets and clothing may remain effective if pyrethroids maintain their repellent effect. In this study, we demonstrated that several pyrethroids maintain repellent effect against resistant mosquitoes, implying that pyrethroid-treated clothing could remain an important tool for personal protection against mosquito-borne infection.

Unexpectedly, we were not able to detect any statistical difference in mortality between NO and PR mosquitoes exposed to any of the pyrethroids, but this is likely because of extremely low mortality even in the susceptible population. Although the NO mosquitoes used in this study are documented to be pyrethroid-susceptible,[24] it is possible that they are not completely sensitive to the insecticides used here, given that we found *kdr* mutations in a large proportion of the tested mosquitoes and observed surprisingly low mortality after arm-in-cage testing.

In our arm-in-cage tests, untreated cloth itself did not reduce landings by mosquitoes, but based on experiments with pyrethroid-sensitive mosquitoes, it did appear to reduce mosquito biting and feeding, likely through its barrier function. Despite low mortality in all tests, cloth treated with all three pyrethroids exhibited strong effect to reduce number of landings and number of blood meals compared to control, though this difference only reached statistical significance for blood meals (most tests) and for landings by NO mosquitoes exposed to permethrin. Permethrin and deltamethrin appeared to be stronger repellents than etofenprox, though though we lacked power to test this relationship; however, all three insecticides had repellent effect against blood meals compared to untreated cloth or bare arm. The difference suggested by our data corroborates a recent study finding that etofenprox is less protective against blood feeding even in pyrethroid-resistant mosquito populations.[44]

Our results are in agreement with other studies that have shown that resistant mosquitoes are repelled by pyrethroids. One *in vitro* study demonstrated a lack of association between *Aedes aegypti* mortality and contact irritant effect (measured by proportion of mosquitoes fleeing from a treated surface after contact) for a variety of insecticides including several pyrethroids.[21,45] Agossa *et al*. showed that wild *Anopheles* mosquitoes in areas with known pyrethroid resistance were spatially repelled by indoor residual spraying of huts in semi-field conditions, with 22–28% fewer mosquitoes entering pyrethroid-treated huts compared to control huts. Blood-feeding was not significantly different inside the treated huts, but human baits did not sleep under nets or use repellents.[46] Our observation that deltamethrin and etofenprox-treated clothing were not superior to permethrin-treated clothing supports the possibility that permethrin-resistance does not affect repellency.

The repellent effect of permethrin against even pyrethroid-resistant mosquitoes is likely to be most important for personal protective measures such as insecticide-impregnated clothing. The efficacy of permethrin-treated clothing against mosquitoes has been demonstrated in many laboratory studies and short-duration field trials.[4,6,7,44,45,47–60] Another laboratory study showed a similar reduction in landing and blood feeding behavior with permethrin-treated clothing compared to untreated clothing using slightly different methods.[45,51] As in our work, they found little difference in landing and blood feeding between resistant and susceptible mosquitoes. Interestingly, in an arm-in-cage experiment in which the arm was only partially covered by cloth, providing mosquitoes with a choice of bare or clothed skin to land and feed on, they observed that resistant mosquitoes rested longer on permethrin-treated cloth and were less likely to move to more bite-susceptible bare arm than susceptible mosquitoes. They interpreted this as a reduction in repellent effect on resistant mosquitoes; but when the arm was fully covered, they did not see a difference in landing or blood feeding, suggesting that repellent effect is robust for both strains.[45]

Field studies of permethrin-impregnated clothing also support a repellent effect for treated clothing, though by design they are unable to examine differences in effectiveness between pyrethroid-resistant and susceptible mosquitoes. A US-based study in park rangers documented a significant difference in antibody titer to *Aedes spp*. salivary antigen between treated and control groups, suggesting that this clothing did exert a sizable protective effect against mosquito biting.[50] The largest field-based trial of factory-treated clothing found no effect on 5-month dengue incidence of permethrin-impregnated school uniforms in a cohort of 1811 Thai children, but poor quality cloth that permitted rapid washout of insecticide with laundering was a major limitation to this study. However, they did detect a reduction in the number of *Ae*. *aegypti* mosquitoes captured in classrooms randomized to treated clothing during the first month of the study, prior to washout of the permethrin, suggesting that the clothing did have a repellent effect on mosquitoes.[8]

Our study has several limitations. First, we are comparing two mosquito populations which have been bred in the laboratory for different periods of time since egg collection. This difference likely affects the populations’ heterozygosities and possibly their biology and behavior. Second, based on genotyping results, kdr mutations are found frequently in “susceptible” NO mosquitoes, raising questions about the effect of pyrethroids on this population. Although *kdr* mutations associated with pyrethroid resistance were detected even in phenotypically susceptible populations, sequencing confirmed that phenotypically resistant PR mosquitoes were significantly more likely than susceptible NO mosquitoes to have one or both of two *kdr* mutations, 1016I or 1534C. While we did not perform genetic testing on the exact same mosquitoes that were used for arm-in-cage tests, they came from the same population and would be expected to have a similar distribution; however, this limited our ability to correlate specific *kdr* mutations or combinations of mutations with mortality or with landing and feeding behavior. Second, we did not look for other mutations in the VGSC gene, which might have affected resistance to or repellency by the pyrethroids used in this study. Third, we were unable to examine another common mode of resistance, upregulation of detoxifying enzymes such as CYP 450 enzymes,[61] and thus cannot determine how any insecticide resistance due to metabolic changes affects the repellent activity of pyrethroids. In at least one study, however, *kdr* mutations were the more important mechanism of resistance to pyrethroids in adult *Ae*. *aegypti*.[13] Finally, limited sample size restricts our ability to make robust statistical inferences.

Dengue incidence has increased since 2005 despite economic development and vector control efforts.[62] Recent Zika and chikungunya virus epidemics have demonstrated that new arboviral threats are likely to emerge. Because vaccines are not yet widely available for most *Aedes*-transmitted infections and their treatment is supportive, public health institutions will continue to rely on vector control activities to limit arboviral disease transmission. Increasing prevalence of pyrethroid resistance in mosquitoes threatens the effectiveness of vector control activities worldwide. We examined the repellent effect of pyrethroid-impregnated clothing on resistant and susceptible strains of *Ae*. *aegypti*. Although there was not a statistically significant, the magnitude of the repellent effect against landing, blood meals, and blood meals per landing was greater for the resistant mosquitoes. These findings provide evidence that even in the absence of an insecticidal or knockdown effect, pyrethroid-treated cloth can protect against mosquito bites and disease transmission. Both susceptible NO and resistant PR mosquitoes were repelled by treated cloth, implying that for person protection, pyrethroid-impregnated cloth for use in bed nets and clothing will likely remain efficacious even in areas where pyrethroid-resistant mosquitoes are abundant. All in all, our data suggest that LLPI clothing may play an important role in personal protection against mosquito-borne diseases.

## Supporting information

S1 Table Data for *kdr* genotyping of *Aedes aegypti* mosquitoes. The first tab (Puerto Rico) provides results from the pyrethroid-resistant Puerto Rico strain. The second tab (New Orleans) provides results from the pyrethroid-sensitive New Orleans strain.
(XLSX)

S2 Table Data for arm-in-cage entomological studies. Provides data from arm-in-cage trials.
(XLSX)
